# Exploring Extended-Spectrum Beta-Lactamase (ESBL)-Producing *Escherichia coli* in Food-Producing Animals and Animal-Derived Foods

**DOI:** 10.3390/pathogens13040346

**Published:** 2024-04-22

**Authors:** Laryssa Freitas Ribeiro, Natália Maramarque Nespolo, Gabriel Augusto Marques Rossi, John Morris Fairbrother

**Affiliations:** 1 Mário Palmério University Center (UniFucamp), Av. Brasil Oeste, s/n, Jardim Zenith, Monte Carmelo 38500-000, Minas Gerais State, Brazil; laryssaribeiro84@gmail.com; 2 Federal University of São Carlos (UFSCar), Rod. Washington Luís, s/n—Monjolinho, São Carlos 13565-905, São Paulo State, Brazil; nnespolo@ufscar.br; 3 Department of Veterinary Medicine, University of Vila Velha (UVV), Vila Velha 29102-920, Espírito Santo State, Brazil; gabriel.rossi@uvv.br; 4 Département de Pathologie et Microbiologie, Faculté de Médecine Vétérinaire, Université de Montréal, Saint-Hyacinthe, QC J2S 2M2, Canada

**Keywords:** animal-derived foods, antimicrobial resistance, enterobacteria, food safety, pathogens, public health

## Abstract

Antimicrobials serve as crucial treatments in both veterinary and human medicine, aiding in the control and prevention of infectious diseases. However, their misuse or overuse has led to the emergence of antimicrobial resistance, posing a significant threat to public health. This review focuses on extended-spectrum beta-lactamase (ESBL)-producing *Escherichia coli* in animals and their associated food products, which contribute to the proliferation of antimicrobial-resistant strains. Recent research has highlighted the presence of ESBL-producing *E. coli* in animals and animal-derived foods, with some studies indicating genetic similarities between these isolates and those found in human infections. This underscores the urgent need to address antimicrobial resistance as a pressing public health issue. More comprehensive studies are required to understand the evolving landscape of ESBLs and to develop strategic public health policies grounded in the One Health approach, aiming to control and mitigate their prevalence effectively.

## 1. Introduction

Infectious diseases caused by bacteria have long posed significant challenges to societies, not only due to their rapid and widespread transmission but also because of their high rates of mortality and morbidity. The treatment of many of these infections often relies on the therapeutic application of antimicrobials [1]. Antimicrobials serve as vital tools in both veterinary and human medicine, playing key roles in the treatment, control, and prevention of infectious diseases. However, the excessive and repeated use of antimicrobials can lead to unforeseen adverse consequences, such as the development of antimicrobial resistance (AMR) among bacteria, particularly towards modern beta-lactam antimicrobials [2,3]. Extended-spectrum beta-lactamases (ESBLs) are enzymes produced by certain bacteria capable of hydrolyzing extended-spectrum cephalosporins [4,5]. Consequently, they exhibit ineffectiveness against beta-lactam antimicrobials such as ceftazidime, ceftriaxone, cefotaxime, and oxyimino-monobactam. These enzymes may either be inherent to the organism, carried on bacterial chromosomes, or plasmid-mediated, with the potential for conjugation between bacterial populations [6].

ESBLs encompass a range of types, with the most prevalent being the SHV, TEM, and CTX-M [7]. Other noteworthy clinically significant types include VEB, PER, BEL-1, BES-1, SFO-1, TLA, and IBC [8]. These enzymes are primarily synthesized by Gram-negative organisms of the *Enterobacteriaceae* family, notably *Klebsiella pneumoniae* and *Escherichia coli* [6,9]. Certain pathotypes of *E. coli* have been linked to various intestinal and extraintestinal diseases, including diarrhea, urinary tract infections, septicemia, and neonatal meningitis [10]. Moreover, the escalating antimicrobial resistance among enteric pathogens poses a substantial public health challenge [11].

The aim of this review is to deepen our comprehension of *E. coli* strains producing ESBLs in animals and animal-derived foods, which are key contributors to the emergence of antimicrobial-resistant strains, thus presenting a significant public health concern.

## 2. ESBL-Producing *E. coli* in Farm Animals

*E. coli* assumes a significant role as a bacterial commensal inhabiting the intestinal microbiota of diverse animal species, including human beings. Within this association, *E. coli* maintains a harmonious coexistence with its hosts, typically manifesting as a symbiotic relationship devoid of pathogenicity. However, it is imperative to recognize that *E. coli* also emerges as a prominent etiological agent responsible for instigating a myriad of prevalent bacterial infections afflicting both human and animal populations alike. It is crucial to underscore the nuanced nature of *E. coli* strains, as not all variants exhibit benign characteristics; indeed, certain strains harbor virulence factors capable of eliciting disease manifestations in susceptible hosts, encompassing mammals and avian species alike. This dualistic nature underscores the necessity for comprehensive surveillance and vigilant monitoring of *E. coli* populations, facilitating the prompt identification and containment of pathogenic strains to mitigate the associated public health risks posed by this ubiquitous bacterial species [3,4,12]. Additionally, *E. coli* plays a significant ecological role and can serve as a bioindicator for antimicrobial resistance. As *E. coli* is harbored in the intestinal tract of all animal species used for food production and this bacterium demonstrates great genetic versatility and adaptability to constantly changing environments, it can acquire numerous mechanisms of antimicrobial resistance, such as enzymes encoded by plasmids [3].

The utilization of antimicrobial agents has precipitated the proliferation of antimicrobial-resistant strains of *E. coli* within the commensal microbiota, engendering a potential hazard to human health via the consumption of contaminated foods [13], notably animal-derived foods. Antimicrobial substances are frequently enlisted in animal husbandry practices with the overarching objectives of disease prevention, infection control, and optimization of animal growth rates. Although the practice of employing antimicrobials as growth promoters has been phased out in jurisdictions such as the European Union, the United States of America, and Canada, it continues to persist unabated in various other regions across the globe [14].

The excessive and inappropriate use of antimicrobials has resulted in a surge in antimicrobial-resistant microorganisms [15], contributing to the global challenge of antimicrobial resistance [16], including the emergence of ESBL production. In food animals, particularly among enterobacteria isolated from them, genes associated with ESBL production such as *bla*_TEM_, *bla*_SHV_, and *bla*_CTX-M_ are frequently detected [17]. Recent studies conducted in rural communities in Ecuador have targeted domesticated animals that share close proximity with humans. These investigations unveiled the existence of ESBL-producing *E. coli* strains, which possess considerable pathogenic potential. This discovery has sparked concerns regarding the concurrent presence of these strains in both animal and human populations, as well as the potential transmission of multidrug-resistant (MDR) strains from animals to humans [18]. In another study of ESBL-producing *E. coli*, plasmid analysis showed that the plasmid backbones across various lineages exhibit significant similarity and can be nearly identical among animals such as chickens, cattle, and swine, with similarities to plasmids isolated from humans [19]. However, research from Reunion Island in the Southwest Indian Ocean suggested that animals might not serve as the primary reservoir for ESBL-producing *E. coli* detected in humans on the island. The authors advocated for policy measures aimed at prioritizing the mitigation of human-to-human transmission to prevent infections within the population [20].

The prevalence of antimicrobial resistance in commensal bacteria can serve as a valuable indicator of the selective pressure exerted by antimicrobial agents, offering an early warning sign of the emergence of resistance in pathogens [3]. A comprehensive review of global cattle production revealed the widespread occurrence of intestinal colonization and clinical manifestations due to ESBL-producing *Enterobacteriaceae* (E-ESBLs) among cattle in Europe, the Americas, Asia, Africa, and Oceania. This occurrence in six of the seven major world cattle producers suggests a significant contribution of E-ESBLs to the dissemination of these bacteria and ESBL genes throughout ecosystems. Cattle production serves as a crucial ecological niche for the selection of commensal bacteria harboring antimicrobial resistance traits within the microbiota [21]. In a notably intriguing study, researchers collected twenty-four samples of cattle manure from four farms and isolated ceftiofur or cefotaxime-resistant *E. coli*. Following this, the genomes of 18 isolates were sequenced, and all of them were found to carry at least one β-lactamase *bla* gene, namely TEM-1, TEM-81, CTX-M115, CTX-M15, OXA-1, or CMY-2, and each of them harbored antimicrobial resistance plasmids associated with classic Inc groups. Consequently, it is evident that storing dairy cow manure for a brief period could serve as a reservoir for resistance genes and potentially virulent strains of *E. coli*, posing a risk of dissemination to other bacteria within the same environment. Such a phenomenon, as elucidated by this study, underscores the pressing need for further investigations to comprehensively gauge and mitigate the associated risks [22]. 

Within the dairy production chain, ESBL-producing *E. coli* has been identified across various age groups, with specific strains of certain sequence types (ST) dominant in calves, such as ST362, known for its robust biofilm formation capacity, posing challenges. Recommendations have been made for enhancing hygiene practices to prevent biofilm formation in nipple buckets and for ensuring appropriate antimicrobial usage in treating calf diarrhea [23]. Furthermore, sheep and goats are also recognized as reservoirs for pathogenic *E. coli* strains, posing risks not only to animals but also to humans along the food chain [24,25]. A study examining ESBL-producing *E. coli* in sheep and goats revealed antimicrobial resistance patterns in both healthy and diarrheic animals [26]. Furthermore, there are reports of some strains potentially capable of producing Shiga toxin and carrying genes associated with ESBL production in these animals [27].

The unregulated and excessive use of antimicrobials in aquatic environments has also been identified as a significant driver of antimicrobial resistance, particularly in *E. coli* strains producing ESBLs. Aquatic animals such as fish and shrimp can serve as reservoirs for antimicrobial resistance genes [28,29], which pose a threat to human health through the consumption of raw seafood, thus contributing to a public health crisis [30]. ESBL-producing Enterobacterales have been observed to transfer the *bla*_CTX-M_ gene to *E. coli*, suggesting a role in the transfer to native gut *E. coli* in humans following the ingestion of contaminated fish [31]. 

**Figure 1:**
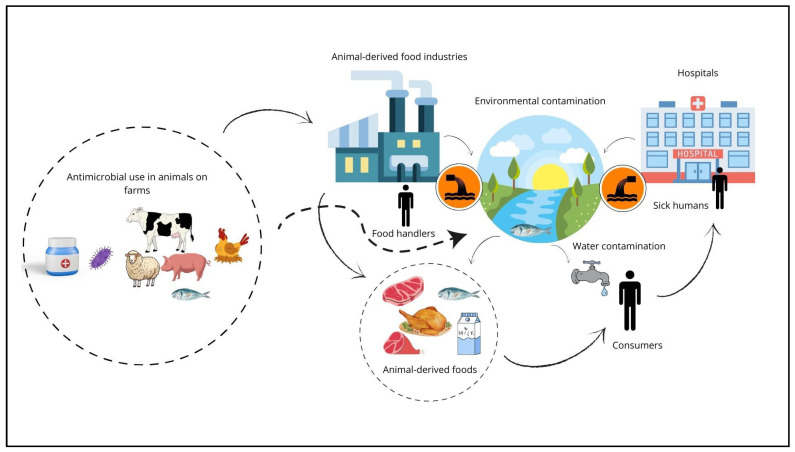
The epidemiology of extended-spectrum beta-lactamase (ESBL)-producing *E. coli* encompasses a multifaceted landscape, elucidating intricate transmission pathways implicated in antimicrobial use within animal husbandry settings, spanning from farms to food industries. Moreover, this complex web extends to encompass the involvement of food handlers, thus amplifying the potential dissemination routes of ESBL-producing *E. coli* strains. Additionally, environmental contamination emerges as a critical facet, with the aquatic ecosystem serving as a reservoir for ESBL dissemination, fueled by a confluence of factors including farm, industrial, and hospital sewage. This comprehensive understanding underscores the far-reaching ramifications of ESBL prevalence and underscores the imperative for holistic intervention strategies targeting various nodes along the transmission continuum to effectively mitigate the burgeoning threat posed by antimicrobial resistance.

Notably, discharge from bovine and poultry slaughterhouses and farms can introduce ESBL-producing microorganisms into aquatic ecosystems, facilitating their dissemination among animal and human populations [32,33,34]. Moreover, improper effluent disposal, particularly when contaminated with human feces, leads to high levels of aquatic contamination by ESBL-producing *E. coli*, underscoring the urgency of preventing the spread of these isolates to human and animal populations [35]. Of particular concern are wastewater discharges from hospitals and healthcare facilities, which have been strongly implicated in the dissemination of ESBL-producing enterobacteria [36,37] (Figure 1). Given these risks, the importance of monitoring freshwater environments for antimicrobial-resistant bacteria or genes, especially in areas susceptible to contamination from human or animal waste, highlights the critical need for enhanced surveillance efforts [38,39]. 

Recent studies have established the widespread presence of *E. coli* producing ESBL and AmpC β-lactamase (pAmpC) in all stages of broiler production systems [40], as well as in swine populations [41]. 

Isolates of multidrug-resistant (MDR) *E. coli* originating from commercial, healthy poultry have been identified, exhibiting a notable prevalence of the IncC and IncFIA plasmids. The majority of these resilient isolates harbored plasmid-mediated resistance genes, *encompassing aph(3″)-Ib*, *aph(6)-Id*, *blaCMY-2*, *floR*, *sul1*, *sul2*, *tet(A)*, and *tet(B)*. Notably, these genetic elements were sourced from *E. coli* strains potentially pathogenic to avian hosts and also belonging to the category of extraintestinal pathogenic *E. coli* (ExPEC). The findings of this investigation suggest that the presence of multiple antimicrobial-resistant *E. coli* isolates retrieved from ostensibly healthy broiler chickens can pose pathogenic threats and serve as reservoirs for antimicrobial resistance determinants [42]. Notably, a high incidence of ESBL-producing bacteria was observed in commercial chickens (*Gallus gallus domesticus*) and, to a lesser extent, in wild birds in Pakistan [43], likely attributable to the use of antimicrobials during chicken rearing. Conventional chicken farms that employ antimicrobial treatments exhibit elevated levels of ESBL *E. coli* compared to organic farms. This discrepancy may be due to the absence of antimicrobial interventions in organic farming practices, coupled with outdoor exposure that minimizes contact with litter, potentially harboring resistant bacteria [44]. In Canada, there has been a notable decrease in the occurrence of ESBL-producing *E. coli* in animals, food, and humans. This decline, observed between 2012 and 2017, is believed to be influenced by a measure implemented by the poultry industry, which banned the preventive application of ceftiofur in chickens in 2014 [45]. Furusawa et al. (2024) have proposed, based on modeling studies in poultry farms, that reducing the presence of ESBL-producing *E. coli* in parent stock and broiler farm environments to less than one percent through improved farm management could effectively reduce their subsequent prevalence in humans [46].

The administration of antimicrobials during the early stages of pig farming has been linked to the detection of ESBL-producing *E. coli* during the final fattening stages, thereby heightening the risk of transmission to humans through the pork production chain. In the European Union, there has been an increasing trend in the prevalence of ESBL-producing *E. coli* in fattening pigs in the last decade, although differences in occurrence between different countries exist due to distinct approaches to the use of antimicrobials in pork production across countries [47,48]. Additionally, ESBL-producing commensal *E. coli* with potential ExPEC pathogenicity have been identified in healthy pigs, underscoring the importance of prevention and transmission reduction efforts from a One Health perspective [49]. 

## 3. ESBL-Producing *E. coli* in Animal-Derived Foods

Food contamination by pathogenic microorganisms poses a significant public health threat and results in substantial economic losses globally. The presence of *E. coli* in food serves as an indicator of potential fecal contamination and the presence of other enteropathogenic bacteria. In addition, certain groups of *E. coli* are inherently pathogenic and can be transmitted through food, and even small quantities can pose significant risks, particularly under conducive environmental conditions that promote their proliferation.

As mentioned above, antimicrobial resistance is widely observed among commensal bacteria from food-producing animals worldwide, raising concerns regarding the impact of antimicrobial usage in animals and the potential transmission of these resistant bacteria to humans through the food chain [3]. The use of antimicrobials critically important in human medicine in food-producing animals has been linked to the emergence of new strains of resistant bacteria, including strains of multidrug-resistant foodborne bacteria such as ESBL-producing *E. coli* [3]. Extensive antimicrobial usage has been documented across various food-producing animals, particularly involving tetracyclines, fluoroquinolones, β-lactams, and aminoglycosides, indicating the widespread presence of AMR in the environment. This, in turn, facilitates the transmission of multidrug-resistant pathogens to humans through the consumption of animal-derived foods [50] and the presence of ESBL-producing bacteria in food waste [51].

Animal-derived products should be acknowledged as a significant source of clinically relevant ESBL-producing bacteria capable of spreading through the food supply chain [52]. The presence of ESBL *E. coli* strains resistant to multiple drugs in street foods derived from animals raises substantial concerns regarding both food safety and public health [53]. Foods originating from animals can harbor antimicrobial-resistant pathogens, a potential source of infections in humans, as demonstrated in a study of ESBL-producing *E. coli* found in retail meats and shrimp at a local market in Vietnam [54]. This underscores the need for the continued monitoring of AMR and public health interventions targeting food safety to control the dissemination of ESBL-producing bacteria in communities.

Moreover, a study conducted in China revealed the contamination of various foods, such as pork, beef, chicken, and shrimp, with ESBL-producing *E. coli* strains [55]. Another study in China identified ESBL-producing *Enterobacteriaceae* predominantly from frozen chicken meat and, to a lesser extent, frozen pork, cold noodles in sauce, cucumber, raw chicken meat, frozen pasta, brine-soaked chicken, and tomato [56]. In samples of commercial chicken meat in Hong Kong, an estimated 80% prevalence of ESBL-producing *E. coli* was reported, with a high frequency of the *bla*_CTX-M-1_ gene [57].

The presence of ESBL-producing *E. coli* has been documented in various food items across different regions, for example, milk samples in India [58], dairy products in Turkey and Mexico [59,60], meats, sushi, and sashimi in Northern Portugal [31,61], and poultry, cattle, swine, and vegetables in Germany [62]. Furthermore, ESBL-producing *E. coli* strains belonging to ST10 have been identified in meats, rectal swab samples from healthy individuals, and even human blood cultures [3]. This ST is a high-risk clonal lineage generally found in humans, and this lineage is also found in animals or food products of animal origin [3]. This evidence highlighted the wide distribution and potential for transmission of ESBL-producing bacteria through various sources, including food and human carriers.

In animal isolates, the prevalent genes linked to this resistance include *bla*_CTX-M-1_, *bla*_CTX-M-2_, *bla*_CTX-M-14_, *bla*_CTX-M-15_, *bla*_TEM-52_, and *bla*_SHV-12_. Additionally, genes conferring resistance to quinolones, aminoglycosides, macrolides, tetracyclines, sulfonamides, trimethoprim, and chloramphenicol have been found in association with *bla*_CTX-M_-containing plasmids. The successful combinations of these genetic elements, along with the co-occurrence of *bla*_CTX-M_ genes with other resistance determinants, likely played a role in the widespread dissemination of CTX-M enzymes, contributing to the current uncontrolled pandemic scenario [3]. When examining the global distribution of genes associated with resistance to extended-spectrum cephalosporins, CTX-M-15 and CTX-M-14 stand out as being the most important. They are widely distributed in both food-producing animals and humans and are frequently detected in clinically important pathogens. Overall, among food-producing animals, the most commonly reported genes encode for CTX-M-type enzymes, including CTX-M-1, -2, -9, -14, -15, -32, and -55. Following CTX-M enzymes, SHV-12 and TEM-52 ESBLs are also frequently observed [3].

The findings from various studies indicate that consumers could potentially be exposed to ESBL-producing *E. coli* through multiple food chains. For instance, Ye et al. (2018) showed that various retail foods can serve as reservoirs for the spread of β-lactam resistance, with resistance genes potentially being transmitted to humans through the food chain [56]. These results, combined with data collected from animal production and human populations in other studies, enable a more comprehensive analysis of foodborne pathways. This analysis should take into consideration the transmission of ESBL-producing bacteria from livestock populations to food at retail establishments and ultimately to consumers.

The involvement of humans as potential sources of contamination of animal-origin products by ESBL-producing *E. coli* cannot be ignored, as several studies have revealed the detection of food handlers carrying isolates with this potential. In Kuwait, a study found that 18.8% of *E. coli* isolates from food handlers could produce ESBL [63]. Similarly, in Nigeria, a high frequency of ESBL-producing *E. coli* was detected on the hands of retailers (40.6%) and butchers (75.0%) who handled pork [64]. Comparable findings were reported among food handlers in Ethiopia [65,66] and Egypt [67]. In Tunisia, stool samples collected from 2135 food handlers revealed that 419 (19.63%) *E. coli* isolates were considered ESBL producers, with a notable prevalence of strains belonging to ST131. The most common genes found in these cases were the *bla*_CTX-M-15_ gene (60%), the *bla*_CTX-M-1_ gene (16.8%), and the *bla*_CTX-M-27_ gene (12.8%) [68]. 

Thus, it is evident that not only animals but also food handlers themselves can serve as potential sources of contamination for animal-origin foods traded globally. Consequently, rigorous hygienic practices must be adopted throughout the entire food production chain to prevent the spread of ESBL-producing *E. coli* and minimize the impact on public health. 

## 4. ESBL-Producing *E. coli* and Public Health

Antimicrobial-resistant bacteria pose a significant global challenge, underscoring the urgent need for surveillance and effective preventive strategies. According to the World Health Organization [16], the spread of antimicrobial-resistant bacteria has escalated across all regions worldwide, emerging as a leading cause of mortality on a global scale. Clinical and public health concerns stemming from multidrug-resistant (MDR) bacterial infections have intensified in recent years, with ESBL-producing *E. coli* being prevalent in animals. The risk of transmission of ESBL-encoding genes to humans has emerged as a significant issue. A study in Europe, as cited by Ramos et al. (2020), documented approximately 300,000 infections and 9000 deaths attributed to ESBL-producing *E. coli* [3]. 

Globally, there has been an eightfold increase in the prevalence of ESBL-producing *E. coli* intestinal carriage within communities over the past two decades [69]. Infections caused by these ESBL-producing microorganisms are associated with elevated mortality rates [70] and prolonged hospitalization periods, particularly in cases of bloodstream infections originating from the urinary tract [71]. Furthermore, it is believed that the COVID-19 pandemic may have contributed to a heightened dissemination of ESBL-producing Enterobacteriales [72].

In the current landscape, CTX-M enzymes have emerged as the most prevalent type of ESBLs, with a significant proportion of ESBL-producing isolates being *E. coli* strains expressing CTX-M β-lactamases. Notably, these strains have transitioned from hospital settings to community environments, raising substantial concerns about their pandemic spread. Of worry is the dissemination of CTX-M-15-producing *E. coli* belonging to ST131, which falls within the highly virulent phylogenetic group B2 of ExPEC. This clonal lineage is associated with a range of severe infections, including urinary tract infections, bacteremia, urinary sepsis, and neonatal sepsis [3]. 

The prevalence of food-producing animals carrying *E. coli* strains producing CTX-M-type ESBLs has substantially increased, prompting inquiries into the potential role of animals and food as reservoirs for this phenomenon [3]. Furthermore, the zoonotic potential of ESBL-producing bacteria, coupled with the co-resistance often observed with CTX-M expression, poses direct implications for human health by limiting treatment options. Hence, careful consideration of the use of fluoroquinolones, colistin, or third- and fourth-generation cephalosporins in animals is essential, given their critical importance in treating systemic or invasive Gram-negative bacterial infections in humans [3].

Numerous studies have documented the global spread of ESBL-producing *E. coli* within communities. Investigations focusing on chickens and pigs, as well as their corresponding meat products, healthy individuals, and patients suffering from urinary tract infections (UTIs), have consistently revealed resistant *E. coli* isolates. These isolates exhibit resistance to antimicrobials such as ampicillin, streptomycin, and tetracycline. Notably, the resistance patterns observed in isolates from UTI patients closely resemble those found in various types of meat and animals. These findings underscore the role of food and animals as significant sources of antimicrobial-resistant pathogens, posing a risk to both UTI patients and the broader community [73].

The food reservoir encompasses both food-producing animals and food products, with a wide array of AMR plasmids documented. Specific combinations of AMR genes and plasmids were found to be more prevalent in food and food-producing animals. These plasmids serve as significant carriers of ESBL/AmpC-, carbapenemase-, and colistin-resistance genes, including types IncF, IncI1, IncN, and IncHI1. Moreover, subtypes of IncF plasmids like F2:A-:B-, F33:A-:B-, or F31:A4:B- have been extensively distributed across samples from foods and food-producing animals [74]. Plasmid-mediated ESBL and AmpC β-lactamase (ESBL/pAmpC)-producing bacteria have been detected across all levels of the broiler production chain. Instances of ESBL *E. coli* urinary tract infections (UTIs) in women who had not been exposed to antimicrobials were linked to isolates sharing the same clonal lineage found in both urine and fecal samples [40]. Additionally, ESBL-producing *E. coli* strains isolated from swine and healthy human hosts may act as reservoirs for community-acquired antimicrobial resistance [41]. These findings emphasize the potential transmission of foodborne contamination to humans, highlighting the importance of minimizing transmission between hosts to control the spread of ESBL-producing *E. coli* in community settings. Enhanced monitoring of ESBL-*E. coli* epidemiology in food, animals, and humans is imperative [74].

The emergence of ESBL-producing strains is directly correlated with the widespread use of beta-lactam antimicrobial agents. Therefore, measures to control the overuse of third-generation cephalosporins are crucial to mitigating the emergence of drug-resistant strains [54]. Moreover, efforts to develop new antimicrobials capable of combating the rise in antimicrobial-resistant organisms are being actively pursued [75]. Preventing the dissemination of such strains may necessitate the implementation of novel therapeutic and public health strategies [3]. The high prevalence of ESBL-producing *E. coli*, along with their resistance genes and epidemiologically significant clonal lineages found in humans, animals, and environments, underscores the importance of implementing comprehensive antimicrobial resistance surveillance within a One Health framework. Such strategies should include safeguarding the integrity of the water supply chain, implementing biosecurity measures on farms, and strengthening infection prevention and control protocols at both household and facility levels [76,77].

## 5. Conclusions

This comprehensive review offers invaluable insights into the prevalence of ESBL-producing *E. coli* in animals and animal-derived foods. By shedding light on their pivotal role in the development of antimicrobial-resistant strains and the resultant public health implications, it serves as a crucial wake-up call to the escalating threat posed by antimicrobial resistance—a pressing issue in the realm of public health. Thus, it underscores the imperative for vigilant surveillance of ESBL prevalence trends and the adoption of tailored public health interventions. These measures are indispensable for proactively addressing the challenges posed by antimicrobial resistance, thereby facilitating effective risk mitigation strategies and the containment of its spread.
